# Metformin‐Associated Lactic Acidosis During Docetaxel Therapy for Castration‐Resistant Prostate Cancer: A Case Report

**DOI:** 10.1002/iju5.70055

**Published:** 2025-06-16

**Authors:** Y. Inoue, A. Niimi, T. Kudo, U. Yoshizaki, Y. Sato, H. Kume

**Affiliations:** ^1^ Department of Urology The New Tokyo Hospital Chiba Japan; ^2^ Department of Urology Tokyo Metropolitan Bokutoh Hospital Tokyo Japan; ^3^ Department of Urology, Graduate School of Medicine The University of Tokyo Tokyo Japan; ^4^ Department of Urology National Center for Global Health and Medicine Tokyo Japan

**Keywords:** castration‐resistant prostate cancer, chemotherapy, metformin‐associated lactic acidosis, patient education, sick‐day management

## Abstract

**Introduction:**

Docetaxel is a key treatment for castration‐resistant prostate cancer and is administered with prednisolone, which increases the risk of steroid‐induced diabetes. Its myelosuppressive effect also increases vulnerability to febrile neutropenia. Metformin is widely used for glycemic control; however, elderly cancer patients are particularly susceptible to metformin‐associated lactic acidosis, necessitating careful management of sick‐day and febrile neutropenia during chemotherapy.

**Case Presentation:**

We report a 70‐year‐old male with castration‐resistant prostate cancer and diabetes mellitus who developed febrile neutropenia on Day 5 following docetaxel initiation. He progressed to shock with severe metabolic acidosis on Day 7 and died despite intensive care, including continuous renal replacement therapy. A retrospective review revealed continued metformin use despite prodromal fatigue and loss of appetite, likely due to impaired judgment.

**Conclusion:**

This case may have involved septic shock, but metformin likely worsened the lactic acidosis. It highlights the need for sick‐day education and monitoring in elderly cancer patients.

**Abbreviations**
CKD: chronic kidney disease
CRPC: castration‐resistant prostate cancer
CRRT: continuous renal replacement therapy
ICU: intensive care unit
MALA: metformin‐associated lactic acidosis
RRT: renal replacement therapy

**Summary**

- Patients undergoing chemotherapy, including docetaxel, often receive steroids, increasing the risk of diabetes.
- While metformin is widely used for the treatment of diabetes, elderly prostate cancer patients are more susceptible to metformin‐associated lactic acidosis.
- Comprehensive patient education on sick‐day management is essential to ensure timely discontinuation and reduce the risk of lactic acidosis.

## 1 Introduction

Docetaxel is a key treatment for castration‐resistant prostate cancer (CRPC) and is administered with prednisolone, which increases the risk of steroid‐induced diabetes. In addition, many prostate cancer patients are elderly and have a higher incidence of diabetes than younger patients. Metformin, a widely used biguanide for type 2 diabetes mellitus, is generally well tolerated but may rarely induce lactic acidosis—a potentially fatal adverse event [1]. Metformin‐associated lactic acidosis (MALA) is defined by the presence of metabolic acidosis (pH < 7.35) and elevated lactate levels (> 5.0 mmol/L) in the presence of metformin use [2]. Risk factors include renal impairment, dehydration, acute illness, and advanced age. Here, we report a case of septic shock which was exacerbated by MALA occurring during docetaxel therapy for CRPC, emphasizing the critical need for metformin discontinuation in high‐risk patients during acute illness.

## 2 Case Presentation

A 70‐year‐old male with a history of diabetes mellitus was diagnosed with prostate cancer (initial PSA: 2167 ng/mL; Gleason score: 4 + 5) accompanied by multiple bone metastases. Following 18 months of combined androgen blockade (CAB) therapy, the disease progressed to CRPC. The patient was then treated with androgen receptor axis‐targeted therapy (ARAT) before being switched to docetaxel therapy (tri‐weekly; 75 mg/m^2^, 125 mg/body). At the initiation of docetaxel therapy, liver function was within normal parameters (AST 28 U/L, ALT 24 U/L, LDH 167 U/L, ALP 49 U/L). Renal function showed mild impairment, with a blood urea nitrogen (BUN) level of 33.2 mg/dL, serum creatinine of 1.32 mg/dL, and an estimated glomerular filtration rate (eGFR) of 41 mL/min/1.73 m^2^. Glycemic control was excellent under metformin therapy, as indicated by an HbA1c of 5.7%. At that time, the patient reported no notable symptoms such as loss of appetite or general fatigue.

**FIGURE 1:**
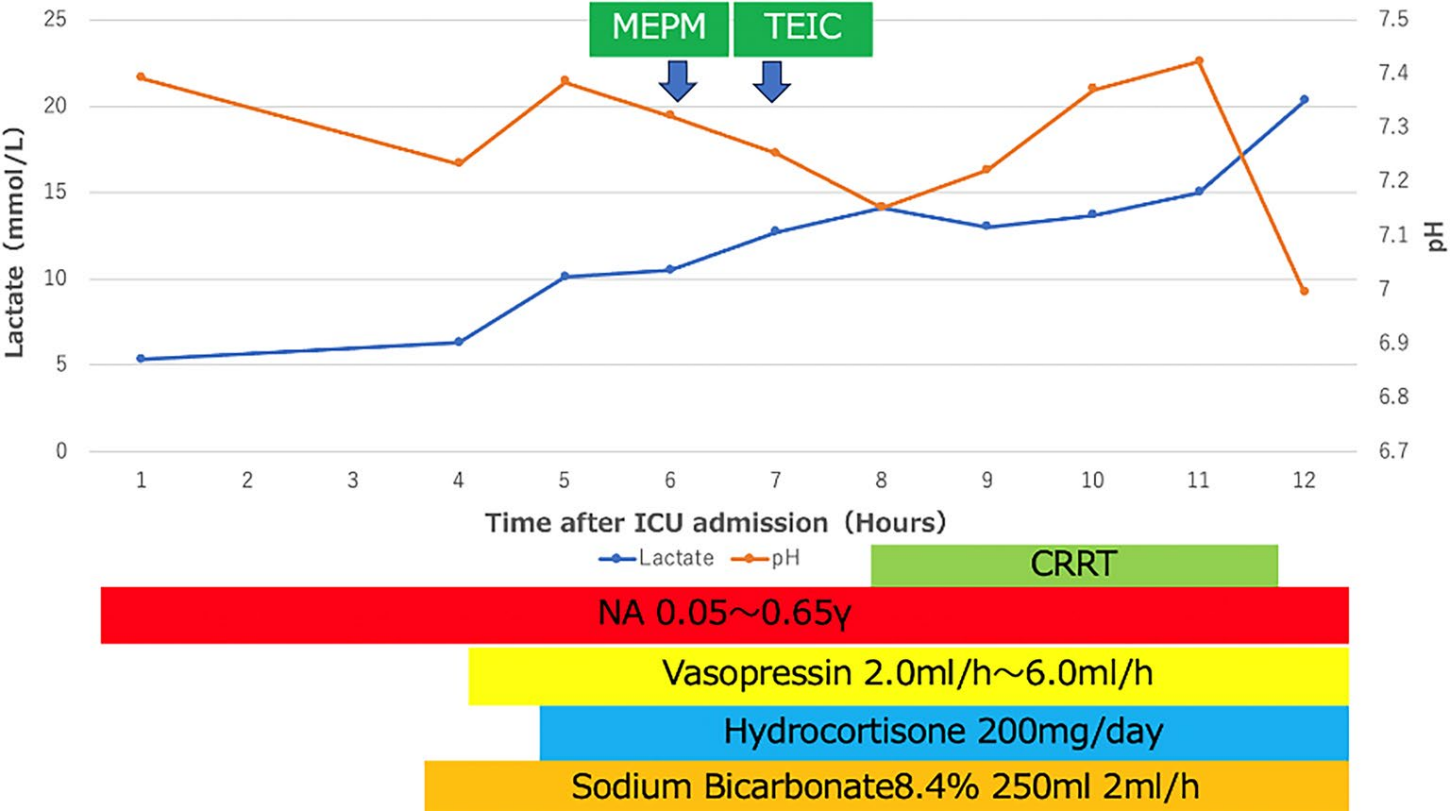
The time course of lactate levels and pH, along with the treatments administered after ICU admission.

On Day 4 post‐docetaxel induction, a reduction in leukocyte count was observed in the absence of overt clinical symptoms; consequently, a long‐acting granulocyte colony‐stimulating factor (G‐CSF) was administered. On Day 5, the patient developed febrile neutropenia (WBC = 1700/μL, Neut = 314/μL). Although computed tomography (CT) did not reveal an obvious source of infection, prophylactic antibiotic treatment with levofloxacin was initiated. No bacteria were detected in the blood, urine, or sputum cultures obtained on the same day, prior to the administration of antibiotics. On the evening of Day6 the patient visited the ER and was admitted to the hospital due to fever and fatigue. Although the Gram staining showed no definitive bacteria, Carbapenem was administered empirically. Despite the white blood cell count recovering to 1800/μL, Neut = 450/μL by Day 7, the patient presented in sudden shock early in the morning and was admitted to the intensive care unit (ICU). Upon admission to the ICU, no significant changes were observed in laboratory parameters other than arterial blood gas analysis. At that time, hepatic function remained preserved, and renal function had even improved following hydration, with laboratory values as follows: AST 33 U/L, ALT 20 U/L, LDH 154 U/L, ALP 36 U/L, BUN 31.5 mg/dL, and Cr 1.17 mg/dL. Arterial blood gas analysis revealed severe metabolic acidosis (pH: 6.995), marked hyperkalemia (K^+^: 7.6 mmol/L), and an elevated lactate level (5.3 mmol/L). Trends in lactate and pH values following ICU admission are depicted in Figure 1. Given the clinical context, MALA was suspected and CRRT was initiated to manage hyperkalemia and acidosis; however, the lactate level remained high (20.3 mmol/L) despite treatment.

The patient's condition rapidly deteriorated, with the development of ventricular fibrillation and pulseless electrical activity. Despite prompt resuscitative efforts, the patient expired on the same day. Subsequent review of his medication history revealed that he had continued taking metformin up to the day of admission despite experiencing severe fatigue and loss of appetite, supporting the diagnosis of metformin‐induced lactic acidosis.

## 3 Discussion

Metformin is commonly associated with gastrointestinal side effects, including diarrhea (40.5%), nausea (15.4%), anorexia (11.8%), and abdominal pain (11.5%) [3]. Although the overall incidence of MALA is low (approximately 30–47 cases per 100 000 patients‐years) [4, 5], its mortality rate is high, reaching up to 50% [6]. Risk factors for MALA include chronic kidney disease (CKD), dehydration, acute illness (sick‐day management issues), alcohol overuse, advanced age, and coexisting cardiovascular, pulmonary, or hepatic disorders, as well as the perioperative state [3, 6].

The underlying mechanism of MALA is thought to involve metformin‐induced inhibition of hepatic gluconeogenesis, particularly the conversion of lactate to glucose, resulting in lactate accumulation. This effect is exacerbated in patients with reduced renal function, as metformin is primarily excreted by the kidneys [7]. Standard pharmacological references describe the plasma half‐life of metformin as approximately 4 h; however, this estimate does not take into account its distribution into slower compartments, such as erythrocytes and mitochondria. In MALA patients, the terminal half‐life has been reported to reach 43–52 h [8], and even in healthy individuals, it may extend to 20–33 h [9]. Radiotracer studies have demonstrated that metformin may persist for more than 24 h in the gastrointestinal tract, liver, and kidneys [10].

In the present case, the patient's eGFR was decreased (41 mL/min/1.73 m^2^), and concurrent dehydration was observed. Therefore, a significant proportion of the total metformin load was likely still circulating at the time of shock onset. Consequently, while sepsis may have contributed to the development of acidosis, the accumulation of metformin likely played a substantial role. Moreover, as observed in this case, acute conditions such as febrile neutropenia and dehydration can precipitate acute kidney injury (AKI), thereby further reducing metformin clearance. This may exacerbate lactic acidosis and lead to a vicious cycle of deterioration [11, 12].

The treatment of MALA focuses on rapid intervention to correct acidosis, enhance metformin clearance, and support hemodynamic stability. Immediate discontinuation of metformin is essential to prevent further lactate accumulation [13]. Intravenous fluid resuscitation with isotonic crystalloids is the first‐line therapy to improve renal perfusion and facilitate lactate clearance. Sodium bicarbonate infusion may be used in cases of severe acidemia (pH < 7.1), though its benefits remain controversial. In severe cases with pH below 7.0 or lactate levels exceeding 15 mmol/L, renal replacement therapy (RRT), such as hemodialysis or continuous renal replacement therapy (CRRT), is the most effective method for removing metformin and correcting acidosis [13, 14]. Supportive care, including vasopressor therapy for hypotension and oxygen supplementation for hypoxia, is necessary for maintaining organ perfusion. Frequent monitoring of blood gases, renal function, and electrolytes is critical to guiding treatment decisions. Early recognition and prompt initiation of these therapeutic measures are essential to improving patient outcomes.

This case demonstrates multiple risk factors that contributed to the development of MALA, including advanced age, docetaxel‐induced loss of appetite, dehydration and febrile neutropenia, acute kidney injury impairing metformin clearance, and impaired judgment due to illness, resulting in continued metformin intake. Despite prior sick‐day education, this case highlights the practical challenges faced by elderly, independent‐living patients in managing medication during sick‐days caused by chemotherapy.

Enhanced patient education, closer monitoring, and caregiver involvement may help prevent similar occurrences [14]. Proper coordination between urologists, oncologists, and endocrinologists is crucial to ensure appropriate metformin management in chemotherapy patients. High‐risk patients, particularly elderly individuals, require reinforced sick‐day management education, with a focus on caregiver support and patient comprehension. Telehealth follow‐ups could facilitate early detection of symptoms necessitating metformin discontinuation and improve adherence to sick‐day management protocols [15, 16].

## 4 Conclusion

This case highlights the critical need for vigilance regarding metformin use during chemotherapy, particularly in elderly patients or those at risk for dehydration and acute kidney injury. Temporary metformin discontinuation during acute illness should be emphasized through enhanced patient education and caregiver involvement. A multidisciplinary approach, involving urologists, oncologists, endocrinologists, and primary care providers, is essential to prevent MALA and reduce its high mortality risk.

## Ethics Statement

Approval number is 0282‐1 (The New Tokyo Hospital Ethical Committee).

## Consent

Written consent was obtained from the patient's relatives.

## Conflicts of Interest

The authors declare no conflicts of interest.
